# Chronic bilateral asynchronous achilles tendon rupture treated using modified whole flexor hallucis longus transfer reconstruction: A case report

**DOI:** 10.1097/MD.0000000000021742

**Published:** 2020-08-28

**Authors:** Xiangfeng Zhang, Feng Ruan, Yongping Wu, Huang Lu

**Affiliations:** a Department of Orthopedic Surgery; b Department of Emergency Medicine, The Second Affiliated Hospital of Zhejiang University School of Medicine, Hangzhou, Zhejiang, China.

**Keywords:** bilateral achilles tendon rupture, flexor hallucis longus tendon transfer

## Abstract

**Introduction:**

Achilles tendon rupture is common, but bilateral ruptures are very rare. Treatment of chronic Achilles tendon rupture is very challenging due tendon retraction and atrophied. We report a case of bilateral asynchronous Achilles tendon rupture patient who was treated with modified minimally invasive whole flexor hallucis longus (FHL) tendon transfer to repair the defects.

**Patient concerns:**

A 52-year-old male farmer presented to our hospital complaining of bilateral heel pain that had disrupted his walking for 6 months. The patient had been misdiagnosed and under-treated for 1 year. Physical examination showed that his plantar flexors were tender and weak, with marked hypotrophy of the calf muscles. Bilateral ankle radiographs of both X-ray and computed topography (CT) revealed no bone injure.

**Diagnosis:**

Magnetic resonance imaging (MRI) indicated a bilateral Achilles tendon rupture. The diagnosis was further confirmed by postoperative histological examination, which revealed Achilles tendonitis accompanied by regional calcification and chondrometaplasia.

**Interventions:**

Surgical reconstruction of the ruptured Achilles tendons was done through a modified minimally invasive whole FHL tendon transfer followed by physiotherapy.

**Outcomes:**

The patient was immobilized in a cast for the next 6 weeks, gradual weight bearing gradually was then encouraged for another 6 weeks, and full weight-bearing started 3 months after surgery. By 6-month postoperation, the patient could walk and jog normally returned to his pre-injury working condition.

**Conclusion:**

Surgical intervention is among the primary treatment of chronic Achilles tendon rupture. However, one of the challenges in its treatment is providing suitable graft for tendon reconstruction. Our case presents a successful reconstruction procedure using less-invasive whole FHL transfer technique. This surgical technique provides satisfactory clinical and functional outcome and can be considered for future therapy.

## 1 Introduction

Achilles tendon rupture is very common. Rupture in the absence of high-energy trauma occurs most often in the fourth to sixth decades of life^[1]^ and this may present with the symptoms of weakened plantar flexion, pain, and abnormal gait. According to one study,^[2]^ 10% to 25% of ambulant patients with acute tendon rupture have been misdiagnosed or undiagnosed. Transition from acute to chronic tendon rupture is around 4 to 6 weeks post-injury.^[3]^ Chronic Achilles tendon rupture often need surgical intervention, however, the operative techniques used in treatment and reconstruction is challenging due to

1. scarring
2. gap defect between the tendons ends and
3. impairment of the associated muscles involved.^[4,5]^

We encountered a patient with chronic bilateral Achilles tendon rupture and performed reconstruction using the minimally invasive modified whole flexor hallucis longus (FHL) tendon transfer technique. Two remaining fibrous scar stumps and a suture anchor were utilized to fix the tendon.

## 2 Patient information

This study was approved by the Ethical committed of the Second Affiliated Hospital School of Medicine Zhejiang University. A 52-year-old male farmer presented to our hospital complaining of bilateral heel pain that had disrupted his walking for 6 months. He reported a history of left heel pain upon sudden running 1 year previously. Limited plantar flexion forced him to visit the hospital, but his condition was not diagnosed. Symptoms were alleviated after 2 weeks of rest, and were able to walk. However, the patient reported feeling similar pain in his right heel under the same conditions 6 months before he presented to our hospital; the source of this pain had also been misdiagnosed.

He had refused steroid injection 1 year previously, as his Achilles tendon-related complaints were not serious at that time. He denied having any other medical condition, such as diabetes mellitus.

## 3 Clinical finding

Physical examination showed that his plantar flexors were tender and weak, with marked hypotrophy of the calf muscles. The patient was not able to stand on the tips of his toes. A diagnosis of chronic bilateral Achilles tendon rupture was suspected.

## 4 Diagnostic assessment

Bilateral ankle radiographs (X-ray and CT) revealed no bone injury, but MRI identified bilateral Achilles tendon rupture. The proximal stumps had re-contracted, and the rupture sites were about 60 mm proximal to the attachment point on both sides.

The diagnosis was further confirmed by postoperative histological examination, which showed Achilles tendonitis accompanied by regional calcification and chondrometaplasia.

## 5 Therapeutic intervention

**Figure 1:**
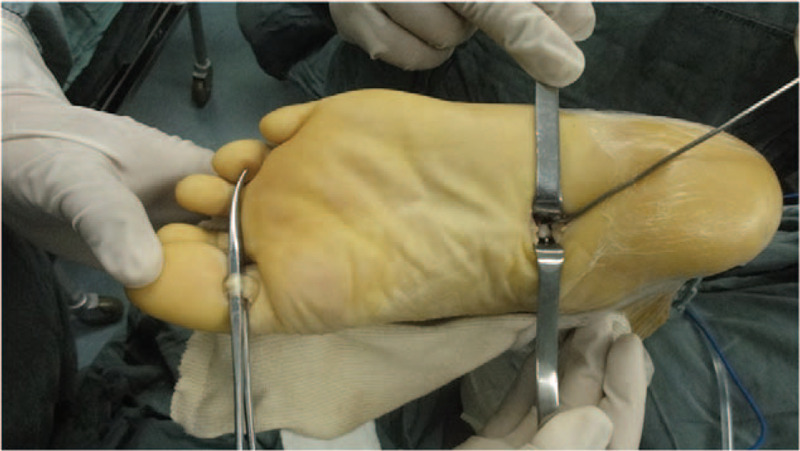
Illustration of surgical procedure. Two incision for whole flexor hallucis longus tendon harvest the “?” shape k-wire was used to separate the flexor hallucis longus tendon and the flexor digitorum longus.

Surgical intervention was used in the treatment of this patient. After the administration of epidural anesthesia, the patient was placed in the prone position, and bilateral thigh tourniquets were applied and inflated sequentially to 300 mm Hg. Ten-centimeter longitudinal incisions were made to explore and expose the stumps and rupture sites of the Achilles tendons and the sural nerves, and avoid any adhesions. After the skin and subcutaneous layer on each side were incised together to form a full-thickness skin flap and the scar tissue was debrided completely, it was sent for histological examination. The final defect was measured with the ankle in plantigrade; the left defect was 6.0 cm and the right was 6.5 cm in length. The entire FHL tendon was harvested in a minimally invasive manner. First, the muscle belly and FHL tendon were exposed, the FHL tendon was retracted, and a K-wire was passed along the tendon groove from the hindfoot to the midfoot. The touchable plantar side of the midfoot was marked, and a 2-cm longitudinal incision was made on the medial aspect of the foot along the marked location (Fig. 1).

The tendon was identified by moving the toe and proximal FHL tendon. The FHL tendon was separated by severing its junction. A third transverse incision was made at the interphalangeal joint of the first toe to separate the distal end of the FHL tendon. Then, the full-length FHL tendon was stretched from the incision to the hindfoot, a length of 21 cm.

A 5.0-mm-diameter suture anchor was fixed 1 cm proximal to the attachment site of the Achilles tendon from the superior-oblique to the posteroinferior side. With the ankle in maximal plantigrade, the proximal part of the FHL tendon was sutured to the anchor.

**Figure 2:**
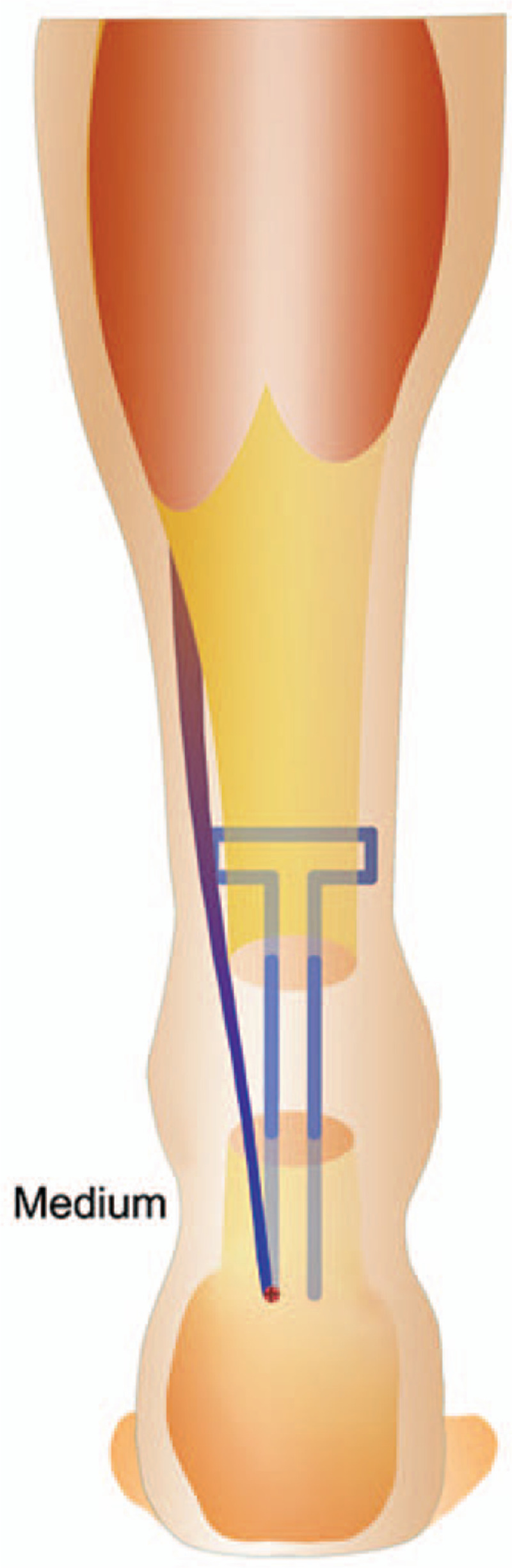
Schematic diagram of the repair technique and suture pattern the anchor suture fixed the FHL on the calcaneus, the free end of the FHL tendon was penetrated with a Kessler's suture.

The FHL tendon was attached with a braided suture, and the stump was penetrated with a Kessler's suture. The free end of the tendon penetrated the distal stump through a drilled channel. The proximal stump was then connected so that penetrated mediolaterally. The proximal stump was penetrated again in the proximodistal direction, and tension was applied across the gap to allow penetration of the distal stump. The FHL tendon was secured to the stump at the entry and exit points (Fig. 2).

**Figure 3:**
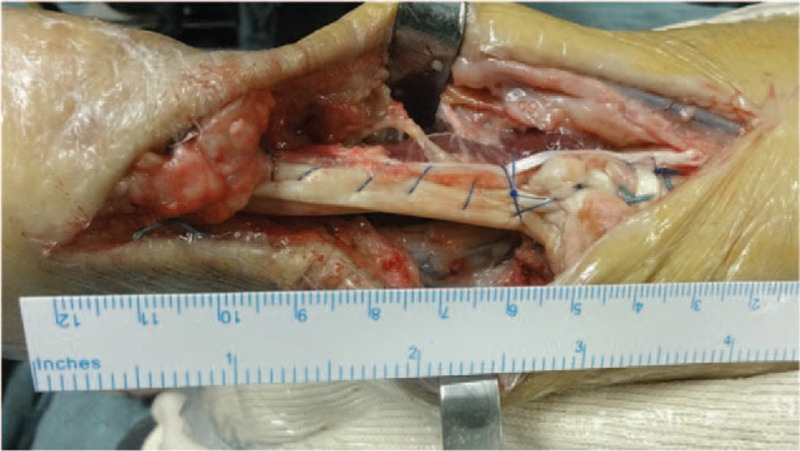
Surgical reconstruction of the tendon. The three tracts of the FHL tendon and the plantaris tendon were sutured and fixed with the FHL muscle belly.

The three tracts of the FHL tendon and the plantaris tendon were sutured and fixed with the FHL muscle belly (Fig. 3). A negative-pressure drainage device was installed, and the subcutaneous layer and skin were sutured.

## 6 Follow-up and outcomes

Antibiotics were administered for 48 h, and the drainage device was then removed. The patient was immobilized in a non–weight-bearing short leg cast for the next 6 weeks. Weight bearing with a prefabricated walking brace was encouraged gradually for another 6 weeks, and full weight-bearing activities were started 3 months after surgery. The patient could walk and jog normally after 6 months and returned to his pre-injury working condition.

## 7 Discussion

Chronic Achilles tendon rupture is defined as an insult with a duration >4 weeks.^[6]^ It is often the result of acute Achilles tendon rupture that is neglected or under-treated. The Achilles tendon ends spread apart and the gastrocnemius–soleus–Achilles tendon complex gradually contracts. Constant pulling and inevitable retraction render end-to-end anastomosis difficult, which is applied to acute injuries, under this circumstance, therapy for chronic Achilles tendon rupture is much more challenging.

The tendon ends were markedly retracted and atrophied in our patient. Given the severe pain that affected his walking, complete debridement of the scar and tissue was necessary. This procedure left a large (>6 cm) gap that had to be bridged; according to Kuwada,^[7]^ a tendon graft should be used in such cases.

Synthetic materials are not used for this type of injury because of the bilateral tender defects and the expense. The use of an autograft or transfer is an alternative method for Achilles tendon reconstruction. Less-invasive^[8]^ and minimally invasive^[9]^ semitendinosus tendon graft augmentation, two turndown flaps and FHL augmentation,^[10]^ and the vascularized gracilis flap^[11]^ or peroneus brevis tendon transfer^[12]^ have been used and shown satisfactory outcomes.

In this case, we chose to use FHL transfer for Achilles tendon reconstruction. Due to proximity of the Achilles tendon, FHL harvesting avoids the need to separate blood vessels and nerves, compared with procedures using the peroneus brevis and flexor digitorum longus tendons. The FHL tendon is functionally the strongest tendon, following the triceps.^[10]^ Moreover, its traction force line resembles that of the Achilles tendon and it shares synchronized contraction with the triceps during the gait cycle. In addition, an FHL graft promotes vascularization of the reconstructed tendon because of its inherent blood vessels.

Most authors have reported FHL tendon harvesting from a midfoot location proximal to Henry's knot^[13]^; however, this method can be insufficient for a large rupture defect. Lui et al^[14]^ reported good results of a full-length FHL transfer performed using a minimally invasive approach. In our case, the total length of the tendon was 21 cm. We used a 5.0-mm-diameter suture anchor to fix the tendon, this decreases the use of the tendon by 1 to 2 cm. In addition, tendon fixation via a suture anchor provides similar fixation strength as drill-hole fixation and has been validated.^[15]^

A minimally invasive approach has been advocated for the midfoot incision to maintain maximum foot function. The passage of a K-wire along the tendon groove from the hindfoot to the midfoot allows determination of the correct location in the skin, with reference to Henry's knot. The proximal plantar approach and minimally invasive incision not only expose the FHL tendon under direct vision, but also contribute to reduced scar formation, thereby maintaining foot function to the maximum extent possible. The tension of the Achilles tendon should be adjusted properly.^[13]^ Graft relaxation or extension is believed to lead to foot deformity or weakened flexion force.

The vascularization of the reconstructed tendon must be optimized to obtain good results. Several factors exemplified by this case are recommended. First, the modified Kessler's suture technique, which increases contact with the tendon stump and promotes vascularization through the channel, should be used. In addition, myodesis of the FHL muscle belly,^[16]^ which also increases vascularity of the tendon, is recommended. Sufficient protection of nerves and blood vessels during the operation should not be ignored.

Reconstruction of a chronically ruptured Achilles tendon is not free from complications. The modified FHL transfer has been reported to reduce toe flexion force and eversion strength of the ankle.^[17]^ Wegrzyn et al^[10]^ reported that all patients exhibited weakness of the hallux interphalangeal joint at 5-year follow-up examinations, but no functional weakness during athletic or daily life activities was detected. In our case, no kinetic or kinematic evaluation of the ankle joint to assess first-toe weakness was performed, but our patient did not complain of this defect. No other complications, such as bleeding, painful scar formation, nerve or blood vessel injury, or foot Varus deformity, occurred in the present case. Our patient could walk and jog normally after 6 months of treatment and returned to his pre-injury working condition.

In conclusion, the treatment of chronic Achilles tendon rupture is very challenging; however, our case presents a successful reconstruction procedure used to treat the tendon rupture. The modified less-invasive whole FHL transfer is an effective surgical technique with good clinical outcome. The results presented here should be supported by additional cases and long-term assessment.

## Acknowledgments

A special acknowledgement goes to the patient on whom the case report is based.

## Author contributions

All authors contributed equally to the writing of this case report.
